# EGCG synergizes the therapeutic effect of irinotecan through enhanced DNA damage in human colorectal cancer cells

**DOI:** 10.1111/jcmm.16718

**Published:** 2021-06-16

**Authors:** Wenbing Wu, Jingying Dong, Hui Gou, Ruiman Geng, Xiaolong Yang, Dan Chen, Bin Xiang, Zhengkun Zhang, Sichong Ren, Lihong Chen, Ji Liu

**Affiliations:** ^1^ Department of Biochemistry and Molecular Biology School of Basic Medical Sciences & Forensic Medicine Sichuan University Chengdu China; ^2^ Department of Biochemistry and Molecular Biology School of Basic Medical Sciences Southwest Medical University Luzhou China; ^3^ Department of Pharmacy The Affiliated Hospital of Southwest Medical University Luzhou China; ^4^ State Key Laboratory of Quality Evaluation of Traditional Chinese Medicine Sichuan Academy of Traditional Chinese Medicine Chengdu China

**Keywords:** apoptosis, autophagy, colorectal cancer, DNA damage, EGCG, irinotecan

## Abstract

Irinotecan is a kind of alkaloid with antitumour activity, but its low solubility and high toxicity limit its application. Epigallocatechin‐3‐gallate (EGCG) is one of the main bioactive components in tea. The epidemiological investigation and animal and cell experiments show that EGCG has a preventive and therapeutic effect on many kinds of tumours. Here, colorectal cancer cells RKO and HCT116 were employed, and the CCK8 proliferation test was used to screen the appropriate concentration of EGCG and irinotecan, and the effects of single and/or combined drugs on migration, invasion, DNA damage, cell cycle and autophagy of tumour cells were investigated. The results showed that EGCG combined with irinotecan (0.5 μmol L^−^) not only had a stronger inhibitory effect on tumour cells than EGCG or irinotecan alone but also prevented tumour cell migration and invasion. EGCG alone did not cause DNA damage in colorectal cancer cells, but its combination with irinotecan could induce S or G2 phase arrest by inhibiting topoisomerase I to cause more extensive DNA damage. EGCG also induced apoptosis by promoting autophagy with irinotecan synergistically. These results indicated that EGCG in combination with irinotecan could be a promising strategy for colorectal cancer.

## 1 INTRODUCTION

Colorectal cancer, as a common malignant tumour of the digestive tract, is a serious threat to human health in the world. According to Globocan 2018 Cancer incidence and mortality statistics provided by the International Agency for Research on Cancer (IARC) of the World Health Organization, among the 18.1 million new cancer cases and 9.6 million cancer deaths worldwide in 2018, colorectal cancer was in the top three.^1^ Surgery is the main treatment for colorectal cancer,^2^ but the 5‐year survival rate is only about 50%. Adjuvant chemotherapy after surgery can effectively reduce the risk of disease recurrence and prolong the overall survival.^3^

Irinotecan (IRI) is used clinically to treat colorectal cancer and small cell carcinoma. Irinotecan is an inhibitor of DNA topoisomerase I (Top1). After entering cells, it does not directly interact with DNA, but covalently binds with the TOP‐DNA complex to form the TOP‐IRI‐DNA complex and stabilize it. The collision of the complex with the advancing replication fork results in a fatal double‐strand break (DSB) that leads to apoptosis.^4^ However, single cancer chemotherapeutic drugs tend to develop resistance or toxicity over time,^5^ and the dose‐limiting toxicity of irinotecan is mainly neutrophil and delayed diarrhoea, other common side effects include vomiting, myelosuppression, alopecia, dyspnoea and fever.^6^ Therefore, the strategy of reducing the side effects and even increasing the antitumour activity of chemotherapeutic drugs by combination therapy has been paid more and more attention.

Some natural active compounds such as EGCG (epigallocatechin‐3‐gallate) and resveratrol have been revealed antitumour activity in vitro or animal experiments.^7^, ^8^ EGCG combined with chemotherapeutic drugs such as Gefitinib^9^ and Bleomycin^10^ can reduce both drug dose and resistance, and it also shows a good synergy effect. Researchers are beginning to try to use these compounds as anticancer adjuvants to enhance the antitumour activity of clinical chemotherapeutic drugs.^11^ Our study focused on the synergistic anti–colorectal cancer effect of EGCG combined with irinotecan, a DNA damage chemotherapeutic agent. The collaborative anti–colorectal cancer effect of EGCG on irinotecan was demonstrated by cell proliferation, migration, invasion and cell cycle, and the molecular mechanism of EGCG was discussed in terms of DNA damage and autophagy pathway.

## 2 MATERIALS AND METHODS

### 2.1 Compounds and reagents

EGCG and irinotecan were purchased from CSN Pharm (Shanghai, China), Dansylcadaverine (MDC) from Sigma‐Aldrich and Cell Counting Kit‐8 (CCK8) from Bimake. DNA Damage Antibody Sampler Kit, CDK4, LC3B, Cyclin D 1 and P‐RBSER807/811 were purchased from CST and Top1, GAPDH, BAX, BCL‐2, PARP, Beclin‐1 and P62 from Proteintech Group. Goat anti‐Rabbit IgG‐FITC antibody was purchased from Hua'an. Annexin V‐FITC Apoptosis assay kit was purchased from Vazyme. A cell cycle and apoptosis assay kit was purchased from Beyotime Biotechnology.

### 2.2 Cell culture

RKO cells were resuscitated from our laboratory, and HCT116 cells were obtained from NuoHe Bio‐Tech; all the cells were grown in RPMI‐1640 medium (Thermo Fisher Scientific) containing 10% (vol/vol) foetal bovine serum (Zhong Qiao Xin Zhou Biotechnology) and added 1% (vol/vol) Penicillin‐Streptomycin (HyClone). Cells were cultured in a Forma Series II Water Jacket CO_2_ Incubator (Thermo Fisher Scientific Instruments) at 37°C in a 5% CO_2_ atmosphere.

### 2.3 Cell proliferation assay

EGCG was dissolved in PBS to form a 21.8 mmol L^−^ reserve liquor and irinotecan in DMSO to 50 mmol L^−^ and then diluted to the corresponding working concentration by RPMI‐1640 serum‐free medium. RKO and HCT116 cells were inoculated in a 96‐well plate and then incubated at 37°C for 60 minutes more with 10% (vol/vol) of CCK8 dissolved in serum‐free medium RPMI‐1640; after 24 hours of irinotecan and/or EGCG treatment, the absorbance at 450nm was measured by iMark (Bio‐Rad Laboratories).

### 2.4 Cell migration assay

Cells were inoculated in a 12‐well plate one day ahead of schedule. When the cells adhered to the wall and converged to 80%‐90%, a 200‐μL pipet tip was used to split the cells. After washed off the cells with PBS, the serum‐free medium was reloaded, and the appropriate concentration of irinotecan and/or EGCG was added. A DM2500 fluorescence microscope (Leica Microsystem) was applied to observe and photograph.

### 2.5 Cell invasion test

The cells in the logarithmic growth phase were replaced by serum‐free medium and then starved for 24 hours. 2 mL Matrigel was thawed in 4°C ahead of time and mixed with 98 mL serum‐free medium, and the concoction was placed in the centre of the upper chamber for 3 hours at 37°C to polymerize. A 600 μL medium containing 20% serum was added to the lower chamber. The upper chamber of Matrigel was carefully placed into the 24‐well. The cells digested by trypsin were cultured for 24 hours with a density of 1.5 × 10^5^ cells/mL, and 300 μL serum‐free cell suspension was laid in the upper chamber.

After incubation, the culture medium was discarded from the upper chamber, then washed with PBS and gently wiped off the remaining cells and Matrigel with a cotton swab. The cells were immersed in methanol solution for 15 minutes, and then, the cells were stained in 0.1% methanol‐soluble crystal violet dye for 15 minutes, and the purple colour was washed off by pure water. The upper chamber was placed in a 37°C incubator to dry, and cells were photographed with a DM2500 fluorescence microscope.

### 2.6 Western blot

Protein was extracted from the treated RKO and HCT116 cells in a 6‐well plate with RIPA lysate (1% protease), separated by 10% SDS‐PAGE and transferred to the NC membrane (Pall Corporation, New York, USA). 0.05% TBST solution with 5% skimmed milk powder was applied to block at room temperature for 1 hour. The membrane was incubated with the corresponding antibody in 0.05% TBST solvent at 4°C overnight, and the HRP‐linked secondary antibody (Signal Antibody) was added at room temperature for 1 hour. ECL substrate (JuHeMei Biotechnology) was utilized and photograph was obtained by Universal Hood II Gel Imaging System (Bio‐Rad Instrument).

### 2.7 Immunofluorescence

According to the CST product instructions, in short, the cells were seeded on a 12‐well plate, fixed with 4% paraformaldehyde for 15 minutes and washed three times with PBS for 5 minutes each. After blocking (1 × PBS/5% normal serum/0.3% Triton^TM^ ×‐100) for 60 minutes, the anti‐γ‐H2AX antibody in dilution buffer (1 × PBS/1% Triton^TM^ ×‐100) was added for 24 hours in 4°C. The cells were rinsed three times and hatched by Goat anti‐Rabbit IgG‐FITC antibody for 1 hour at room temperature. Then, the plate was stained with DAPI for 5 minutes and took photographs with a fluorescence microscope.

### 2.8 Cell cycle distribution measured by flow cytometry

Cells treated in a 12‐well plate are operated according to the manufacturer's instructions. In short, the cells were digested with trypsin and centrifuged at 1500 *g* for 5 minutes to collect in a tube. Add 1 mL pre‐cooled 70% ethanol, fixed at 4°C overnight. The cells were centrifuged at 2000 *g* for 5 minutes and washed by PBS once. A 0.5 mL propidium iodide (PI) solution (25 μL 20 × PI and 10 μL 50 × Rnase A) was added to stain at 37°C for 30 minutes; the flow cytometry (BD Biosciences) was employed for DNA content analysis using the PI channel.

### 2.9 Cell apoptosis detected by flow cytometry

Cells treated in a 12‐well plate are managed according to the manufacturer's instructions. Briefly, the cells were digested with trypsin without EDTA and centrifuged at 2000 *g* for 5 minutes. After rinsing with PBS and centrifuging for 5 minutes at 1000 *g*, Annexin V‐FITC and PI staining were applied for 15 minutes and detected on the flow cytometry through FITC and PI channels.

### 2.10 Autophagy observed by MDC staining

According to the method in literature,^12^ the cells were treated in a 12‐well plate and washed three times with pre‐cooled PBS. 50 μmol L^−^ MDC staining solution was added and incubated at 37°C for 30 minutes. The cells were then rinsed three times with PBS and observed the green fluorescence in the fluorescence microscope. For flow cytometry assay, cells were harvested and stained with MDC followed measurement in flow cytometer by FITC channel.

### 2.11 Data processing and statistical analysis

All the experiments were repeated 3 times, and the data format was mean ± standard deviation. GraphPad Prism 7 (Graph Pad Software) was used for data statistics and mapping. The significance test was performed by one‐way ANOVA and multiple comparison with Bonferroni correction. The data from flow cytometry were processed by FlowJo (TreeStar) or ModFit (Verity Software House).

## 3 RESULTS

### 3.1 EGCG and irinotecan synergistically inhibited the proliferation of colorectal cancer cells

**FIGURE 1:**
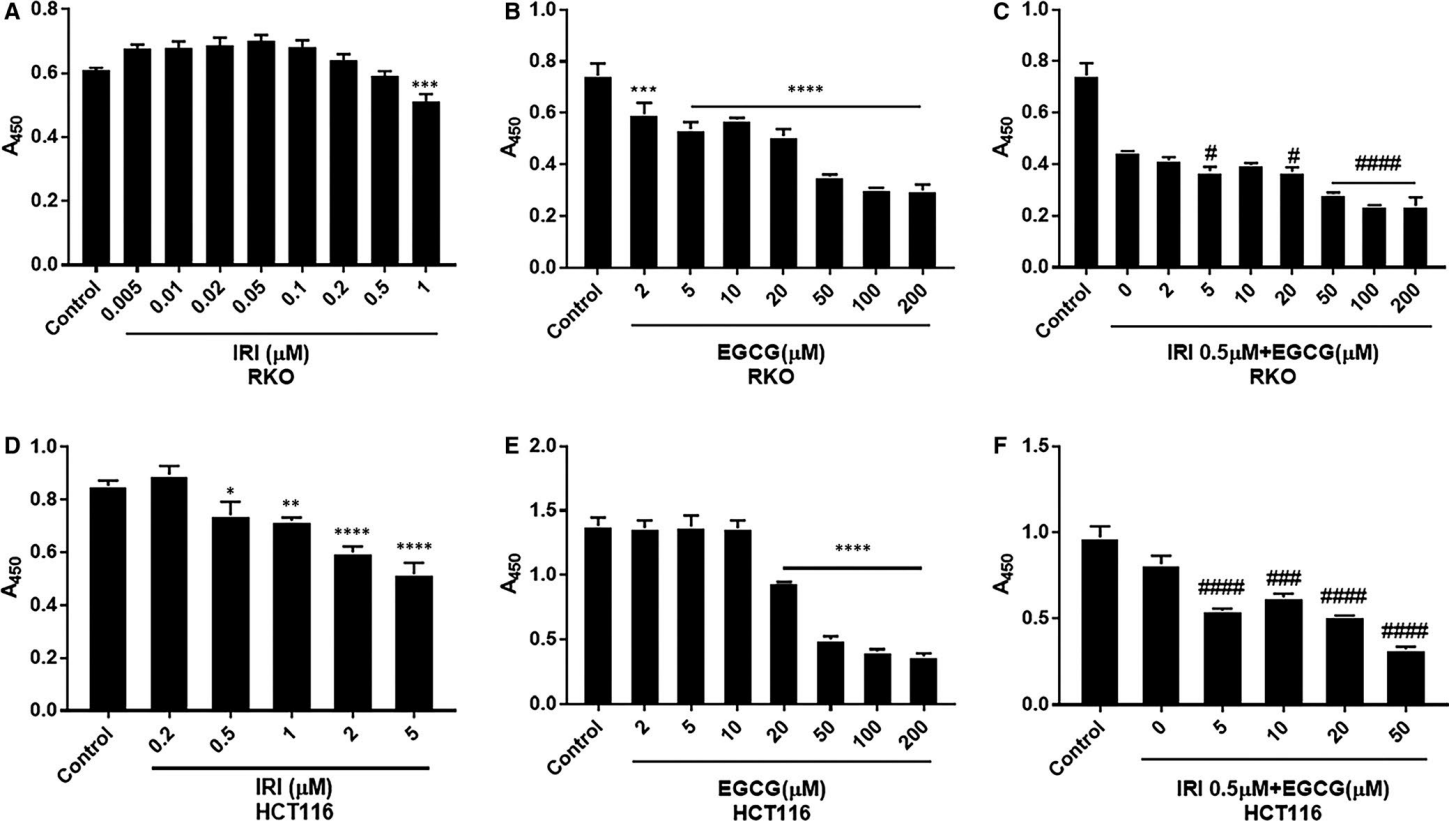
CCK8 assay to detect the proliferation of RKO and HCT116 cells treated with irinotecan and/or EGCG at different concentrations after 24 h. A‐C, are irinotecan, EGCG, 0.5μM irinotecan + EGCG in RKO cells, and D‐F, in HCT116 cells. *P < .05, **P < .01, ***P < .005, ****P < .001 (compared with the control group); ^#^ *P* < .05, ^##^ *P* < .01, ^###^ *P* < .005, ^####^ *P* < .001 (compared with IRI 0 µmol L^−^ group)

To investigate the synergistic effect of EGCG on the antitumour cell proliferation of irinotecan, Cell Counting Kit‐8 was used to determine the 24‐hour inhibition rate of EGCG and/or irinotecan. As shown in Figure 1, irinotecan could prevent RKO and HTC116 cells when the concentration of irinotecan was 0.5 μmol L^−^. Continuous exposure to EGCG also caused a dose‐dependent inhibition on the growth of two cell lines. When the concentration of EGCG was 20 and 50 μmol L^−^, the viability of RKO and HCT116 cells was significantly reduced. The combination index (CI) was calculated by computational model software CompuSyn developed by Chou and Talalay. The results are shown in Tables 1 and 2. The CIs of RKO and HCT116 cells indicated the synergistic effect of irinotecan and EGCG (CI ˃ 1 means antagonistic effect, and CI = 1 additive effect, 0.3 ˂ CI ˂ 0.7 synergistic effect, and CI ˂ 0.3 strong synergistic effect).

**TABLE 1:** Effect and CI of Combination Treatment of Irinotecan and EGCG in RKO Cell

Dose IRI (µmol L^−^)	Dose EGCG (µmol L^−^)	Effect	CI
0.5	10.0	0.46801	0.5737
0.5	20.0	0.50291	0.6497
0.5	50.0	0.61924	0.5568

**TABLE 2:** Effect and CI of Combination Treatment of Irinotecan and EGCG in HCT116 Cell

Dose IRI (µmol L^−^)	Dose EGCG (µmol L^−^)	Effect	CI
0.5	10.0	0.35849	0.3432
0.5	20.0	0.47033	0.3856
0.5	50.0	0.67136	0.4959

### 3.2 EGCG and irinotecan synergistically inhibited the migration and invasion of colorectal cancer cells

**FIGURE 2:**
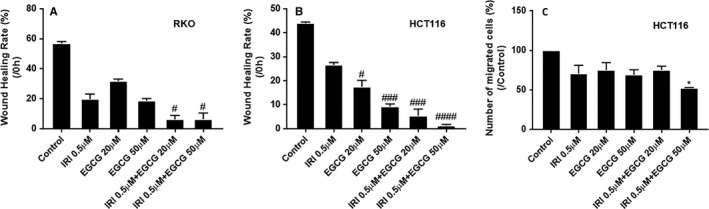
Effect of EGCG and/or irinotecan treatment on migration and invasion of RKO and HCT116 cells for 24 h. Representative graph of the inhibitory effect of irinotecan and/or EGCG treatment on the migration of RKO A, and HCT116 B, cells, and invasion of HCT116 cell C. ^#^ *P* < .05, ^##^ *P* < .01, ^###^ *P* < .005, ^####^ *P* < .001 (compared with IRI 0μM group); *P < .05 (compared with the control group)

The basic principle of the wound healing experiment is to create a blank, cell‐free area in the monolayer of cells on the board. After a period of cultivation, the cells will come into contact with each other again. It is often used to detect the migration ability of tumour cells. As shown in Figure 2 (A, B) and Figure S1, either irinotecan alone, EGCG alone or combination of the two drugs inhibited the migration of both colorectal cancer cells, and the co‐treatment with EGCG and irinotecan led to significantly additive inhibition than either drug alone.

The membrane of Transwell chambers is covered by Matrigel, and the cells cannot pass through it freely. They must secrete matrix metalloproteinase (MMP) to degrade the matrix glue before they can pass through the polycarbonate membrane, which is similar to the situation in vivo. The invasion of HCT116 cells was inhibited by irinotecan alone, EGCG alone or a combination. The higher the concentration of EGCG, the greater the inhibition of cell invasion. When 50 μmol L^−^ EGCG was used in combination with 0.5 μmol L^−^ irinotecan, the cell invasion was significantly prevented (Figure 2 C, Figure S2).

### 3.3 EGCG augmented irinotecan‐induced DNA damage

**FIGURE 3:**
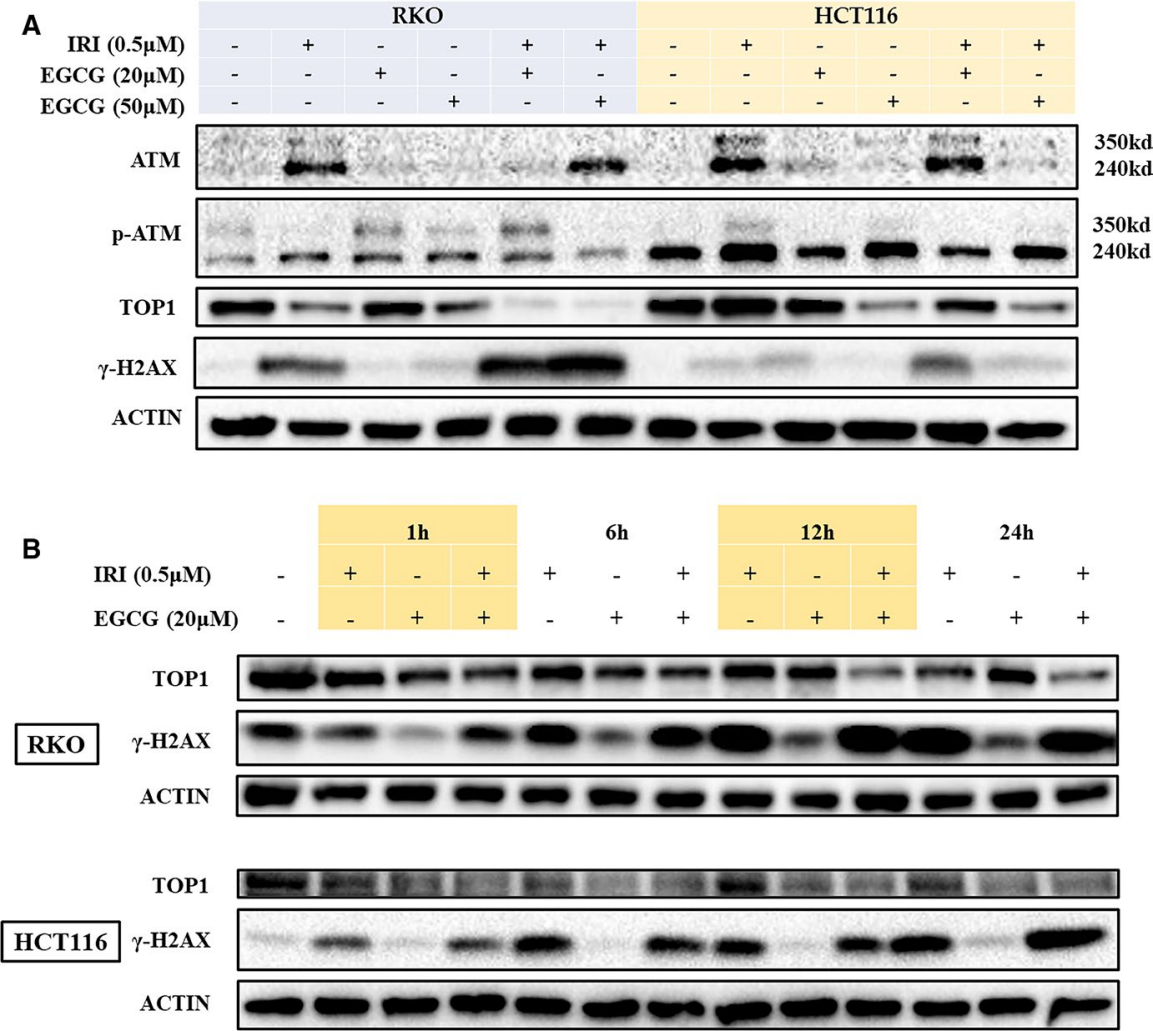
Impact of different concentrations of EGCG and/or irinotecan on DNA damage in RKO and HCT116 cells for 24 h. The WB assay examined the effects of Irinotecan and/or EGCG treatment on the DNA damage response pathway of RKO and HCT116 cells for 24 h A, or 1‐24 h B

Irinotecan and its derivatives, which inhibit topoisomerase I and cause DNA damage, are often used as chemotherapy drugs to treat various cancers.^13^, ^14^ EGCG also induces DNA damage in human normal and cancer cells.^15^, ^16^ Irinotecan increased the DNA damage marker γ‐H2AX in nuclear compared with the control group. EGCG alone did not cause significant DNA damage but combined with irinotecan induced more severe DNA damage. Besides, immunofluorescence results indicated that HCT116 cells with or without irinotecan accumulated in chromatin and formed nuclear fragments, and the nuclear volume increased significantly (Figure S3). WB results showed that the concomitant effect of EGCG on DNA damage may be related to the inhibition of topoisomerase I and increased with time (Figure 3 A, B).

EGCG can increase the cleavage of ataxia telangiectasia‐mutated (ATM) and p‐ATM proteins by co‐treatment with irinotecan. In RKO and HCT116 cells, when the concentration of EGCG was 50 μmol L^−^ and 20 μmol L^−^, respectively, the combination induced more obvious ATM fragmentation than any single drug. During apoptosis, the cleavage of ATM produces a kinase‐free protein that, through its DNA binding ability, inhibits DNA repair and DNA damage signals in a trans‐dominant‐negative manner. Therefore, the expression of the S‐139 phosphorylated product of γ‐H2AX was positively correlated with the disintegration of ATM (Figure 3A).^17^

### 3.4 EGCG combined with irinotecan induced cell cycle arrest

**FIGURE 4:**
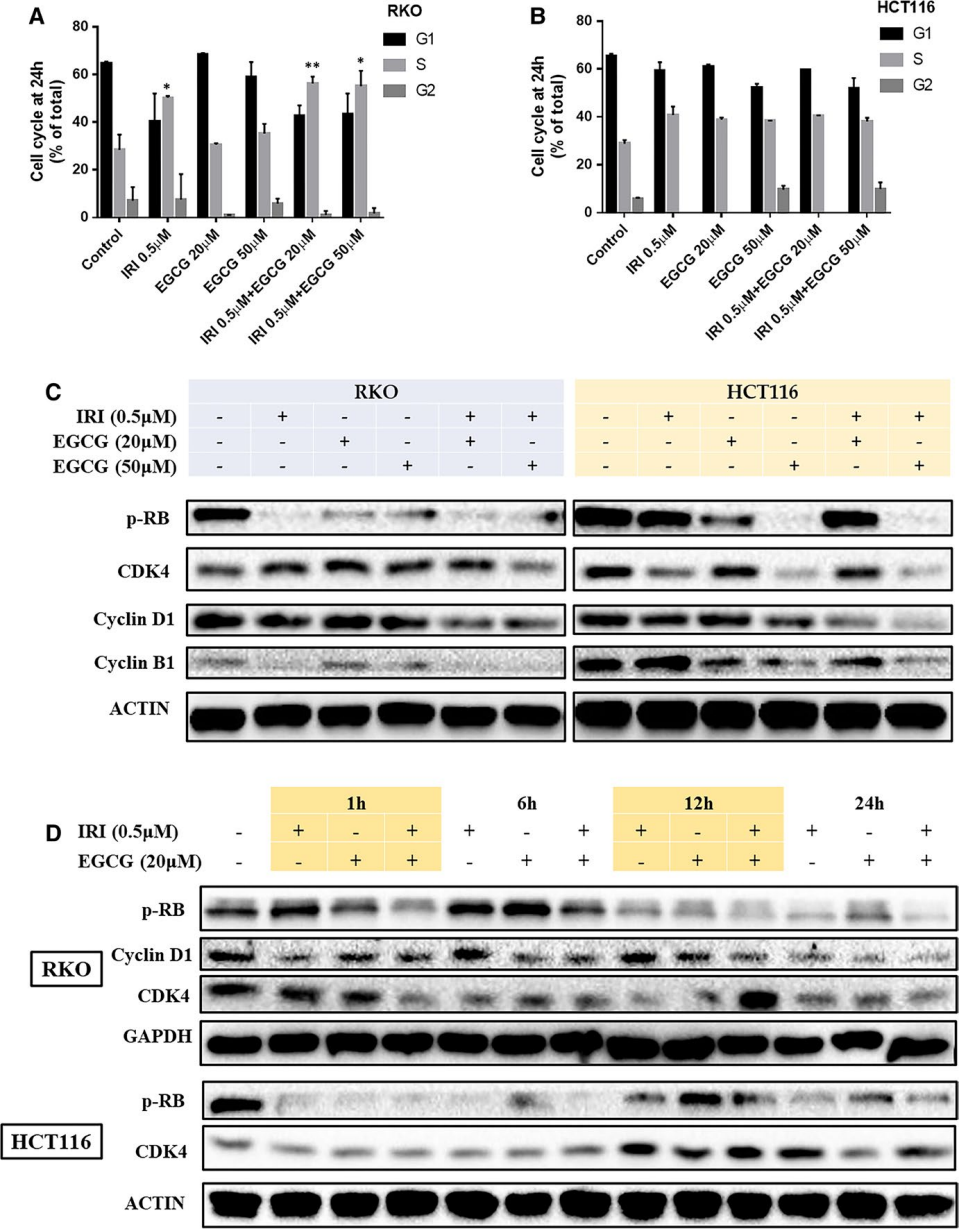
Effects of EGCG on the cell cycle of irinoticam pre‐treated RKO and HCT116. Representative graph of cell cycle distribution in RKO A, and HCT116 B. C and D, are the cell cycle–related proteins detected by the WB experiment. *P < .05, **P < .01 (compared with the control group)

The eukaryotic cell cycle is usually divided into G1, S, G2 and M phases. Cell cycle checkpoints composed of receptors, mediators, transducers and effectors are especially important for cell growth and cell fate. The G1, S and G2 stages are closely related to DNA damage. Therefore, the DNA damage checkpoint is a critical mechanism to maintain the genetic stability in the cell genome, and its function also affects the development of tissues and organs, cell senescence, the occurrence and development of tumours.^18^ As shown in Figure 4 (A, B) and Figure S4, irinotecan can cause S phase cell cycle arrest in RKO and HCT116 cells. When EGCG combined, S phase arrest of RKO cells was promoted, and G2 phase arrest of HCT116 cells was slightly higher than that of control cells. CDK4, Cyclin D 1 and Cyclin B 1 decreased in a dose‐dependent manner with EGCG and time. At the same time, the concomitant treatment of EGCG + irinotecan promoted the phosphorylation of RB (retinoblastoma) protein, with RKO cells showing a peak of phosphorylation at 6h and HCT116 cells lasting up to 24 hours compared with irinotecan alone (Figure 4 C, D).

### 3.5 EGCG elevated the apoptosis of colorectal cancer cells induced by irinotecan through autophagy

**FIGURE 5:**
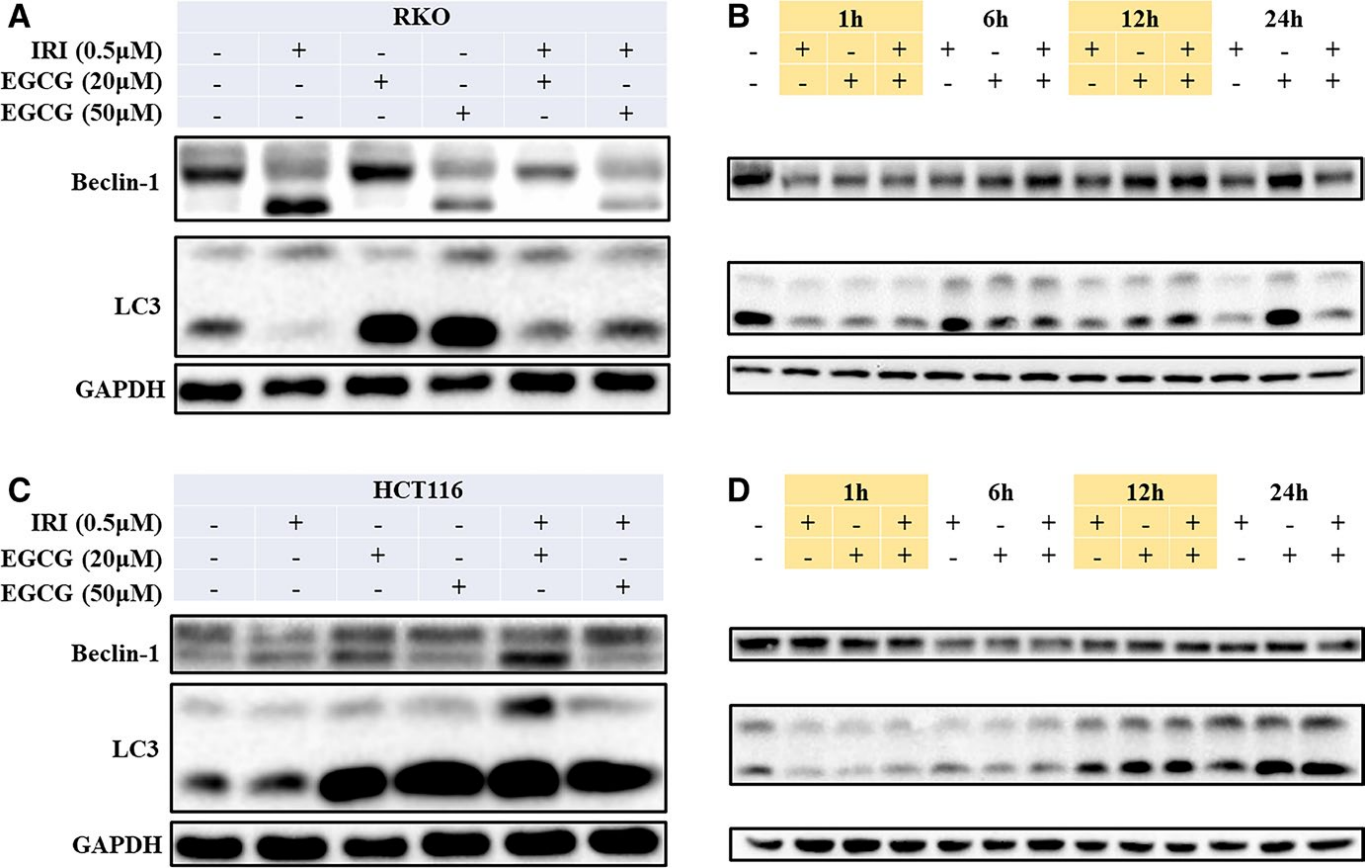
Effects of EGCG on autophagy in RKO and HCT116 cells pre‐treated with irinotecan, WB was applied to detect the expression of autophagy‐related proteins of RKO A, B and HCT116 C, D cells

**FIGURE 6:**
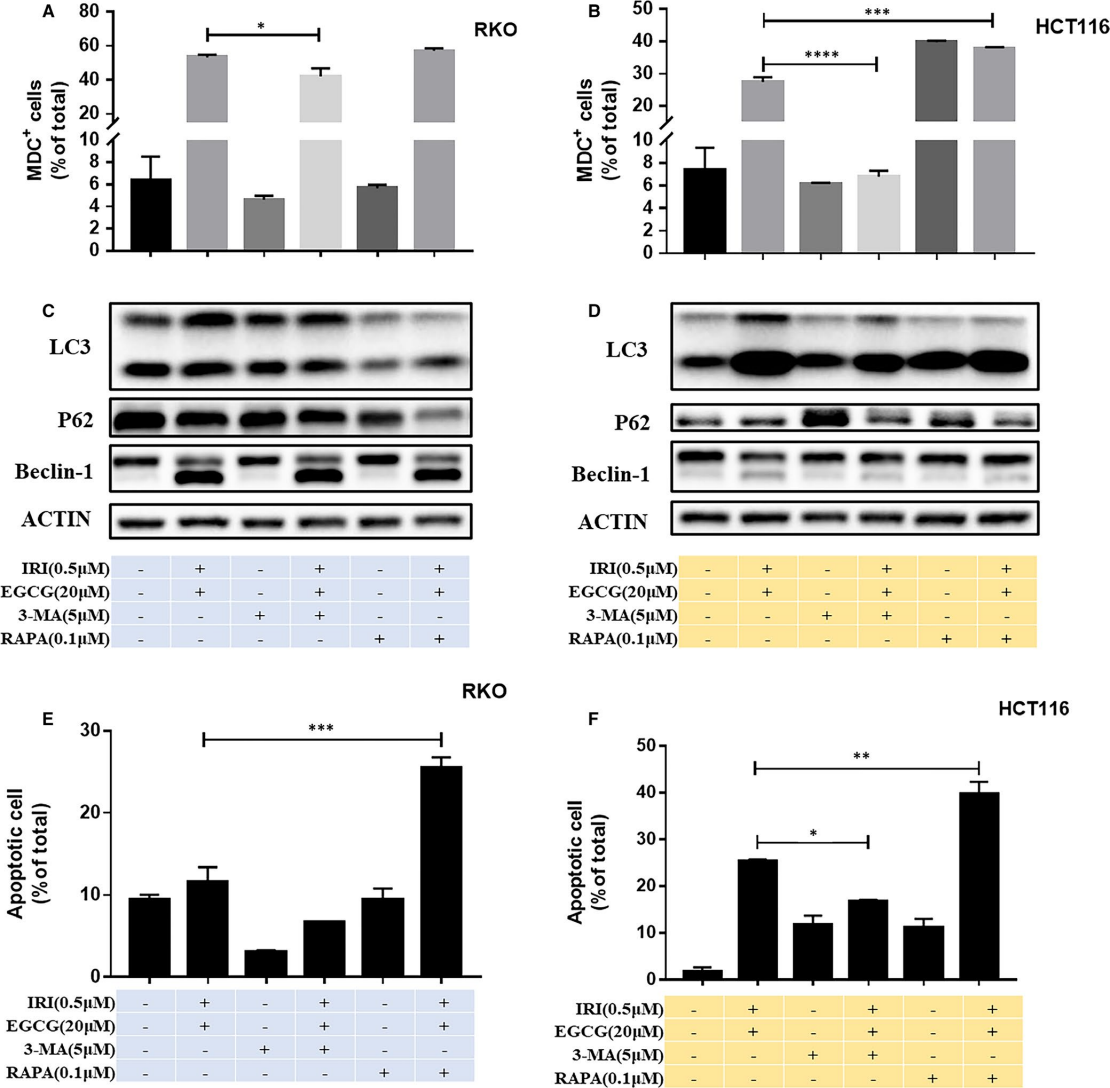
Effects of autophagy on apoptosis of RKO and HCT116 cells induced by EGCG combined with irinotecan. After MDC staining, the representative graph of autophagy changes in RKO A, and HCT116 B, cells was detected by flow cytometry, and the expression of autophagy‐related proteins in RKO C, and HCT116 D, cells were measured by WB. Representative graph of apoptosis rate in RKO E, and HCT116 F, cells, and the data were fitted with FlowJo software. *P < .05,**P < .01,***P < .005,****P < .001

EGCG induced the formation of autophagic vacuoles and the transformation of LC3B II to LC3B I in colorectal cancer cells in a dose‐dependent manner, and the level of autophagy increased gradually with time, reaching the maximum at 24 hours (Figure 5 and Figure S5). When treated with 3‐methyladenine (3‐MA), an inhibitor of autophagy, EGCG combined with irinotecan reduced autophagy and decreased apoptosis. In contrast, autophagy was further promoted and apoptosis increased when treated with autophagy inducer rapamycin (RA) (Figure 6 and Figure S7, Figure S5).

## 4 DISCUSSION

Green tea is one of the most popular and widely consumed beverages in the world, and green tea polyphenols are promising phytochemicals with chemoprophylaxis and chemical protection. EGCG is one of the most abundant catechins in green tea and one of the most studied tea components. Many epidemiological studies have shown that green tea consumption can be a good prevention of various types of cancer and reduce cancer metastasis.^19^, ^20^, ^21^ The anticancer effect of EGCG has been demonstrated in many cell and animal experiments. For example, the combination of EGCG at 0.1 μg/mL and 5‐FU can significantly inhibit the proliferation of head and neck squamous cell carcinoma (HNSCC) cells.^22^ In the concomitant treatment of EGCG and taxol, docetaxel, the synergistic effect even eliminated xenografts of human prostate cancer cell line PC‐3ML in mice.^23^ Our results also suggested that EGCG in combination with irinotecan has a synergistic inhibitory effect on RKO and HCT116 cells. Through cell proliferation, migration and invasion experiments, we demonstrated that EGCG and irinotecan have a synergistic antitumour effect (Figures 1, 2) (Tables 1 and 2).

Similar to irinotecan, EGCG has also been reported to inhibit topoisomerase I in colon cancer cells.^24^ Our experiments demonstrated for the first time that EGCG and irinotecan can synergistically inhibit the activity of topoisomerase I, leading to more extensive DNA damage (Figure 3). ATM is the major inducer of DNA double‐strand breaks (DSBs) and the main conduction factor, which activates downstream proteins and induces DNA damage response.^25^ ATM cleavage appears to occur in parallel with apoptosis, which has also been reported in cisplatin‐induced renal cell injury,^26^ and it does not eliminate the binding activity with the end DNA strand.^17^ Consistent with the reported results, our findings also suggested that EGCG promotes ATM fragmentation and cell apoptosis. (Figure 3 and Figure S7).

The cell cycle is a complex sequence of events in which a cell replicates its DNA and divides to form daughter cells with identical genes. The cycle and its regulation are not only critical for cell growth and reproduction, but also involve many regulatory proteins, such as cyclins and CDKs, interphase oncogenes, tumour suppressor genes and mitotic checkpoint proteins that promote or inhibit this process at various stages of the cell cycle. When cells undergo DNA damage, CDKs are suppressed, and the cell cycle is arrested. Activation of CDK4 and CDK6 affects the early stages of G1; these CDKs bind to Cyclin D and phosphorylate RB, preventing them from binding and inhibiting E2F transcription factors, which are necessary for the transcription of G1/S conversion and promotion of the next phase of the cell cycle.^27^ Our results suggested that EGCG induced S phase or G2 phase arrest by synergizing with irinotecan in promoting DNA damage (Figure 4).

DNA damage can induce autophagy.^28^ DNA damage chemotherapeutic drugs such as camptothecin,^29^ etoposide^30^ and ionizing radiation^31^ have been shown to induce autophagy as well as initiate cell cycle arrest. ATM may be involved in autophagy. When cells are exposed to genotoxic or oxidative compounds, ATM is activated, and the mTORC1 signalling pathway is inhibited to induce autophagy.^32^ The effect of EGCG on autophagy has been studied pre‐clinically, and the role of EGCG induced autophagy in tumour cell death has been reported. Satoh^33^ had observed that EGCG induces human mesothelioma cell death by affecting autophagy. Calgarotto^34^ reported that green tea and quercetin induced autophagy in human leukaemia HL60 cell xenografts.

BCL‐2 interacting protein‐1 (Beclin‐1) is a protein encoded by the BECN1 gene. Beclin‐1 is a mammalian homolog of yeast autophagy‐related gene ATG6 and nematode BEC‐1. This protein interacts with BCL‐2 or class III PI3K proteins and plays a key role in regulating autophagy and cell death.^35^ When human colorectal cancer cells were treated with sublethal chemotherapy drugs such as melphalan and autophagy inducers such as rapamycin, autophagy was converted to apoptosis by cleavage of the key molecule Beclin‐1 induced by Caspase‐8.^36^ Our results showed that EGCG induces autophagy in a dose‐ and time‐dependent manner. Irinotecan alone did not appear to induce autophagy, but it stimulated Beclin‐1 cleavage, and EGCG enhanced autophagy (Figure 5). Concerning the results of apoptosis, together with the impact of autophagy induction and inhibition on apoptosis, we hypothesized that EGCG could turn autophagy into apoptosis by promoting autophagy and Beclin‐1 cleavage, which increased the toxicity of irinotecan in colorectal cancer cells.

## CONFLICTS OF INTEREST

The authors confirm that there are no conflicts of interest.

## AUTHOR CONTRIBUTIONS

**Wenbing Wu:** Conceptualization (equal); Data curation (equal); Formal analysis (equal); Investigation (equal); Methodology (equal); Validation (equal); Writing‐original draft (equal); Writing‐review & editing (equal). **Jingying Dong:** Conceptualization (supporting); Data curation (supporting); Investigation (equal); Methodology (equal); Software (supporting); Visualization (supporting); Writing‐original draft (equal); Writing‐review & editing (equal). **Hui Gou:** Writing‐original draft (supporting). **Ruiman Geng:** Data curation (supporting); Investigation (supporting). **Xiaolong Yang:** Investigation (supporting); Validation (supporting). **Dan Chen:** Investigation (supporting); Software (supporting). **Bin Xiang:** Data curation (equal); Visualization (equal). **Zhengkun Zhang:** Data curation (equal); Software (equal). **Sichong Ren:** Data curation (supporting); Funding acquisition (lead); Software (supporting). **Lihong Chen:** Project administration (equal); Writing‐review & editing (equal). **Ji Liu:** Project administration (equal); Writing‐review & editing (equal).

## Supporting information

Fig S1‐S7

## Data Availability

The data that support the findings of this study are available from the corresponding author upon reasonable request.
