# Supplementary material for: EGCG synergizes the therapeutic effect of irinotecan through enhanced DNA damage in human colorectal cancer cells
Source: J Cell Mol Med. 2021 Jun 16;25(16):7913–21. doi: 10.1111/jcmm.16718 (PMC8358867; doi:10.1111/jcmm.16718)
Supplement: Supplementary file 1 — Fig S1‐S7 [file JCMM-25-7913-s001.docx]

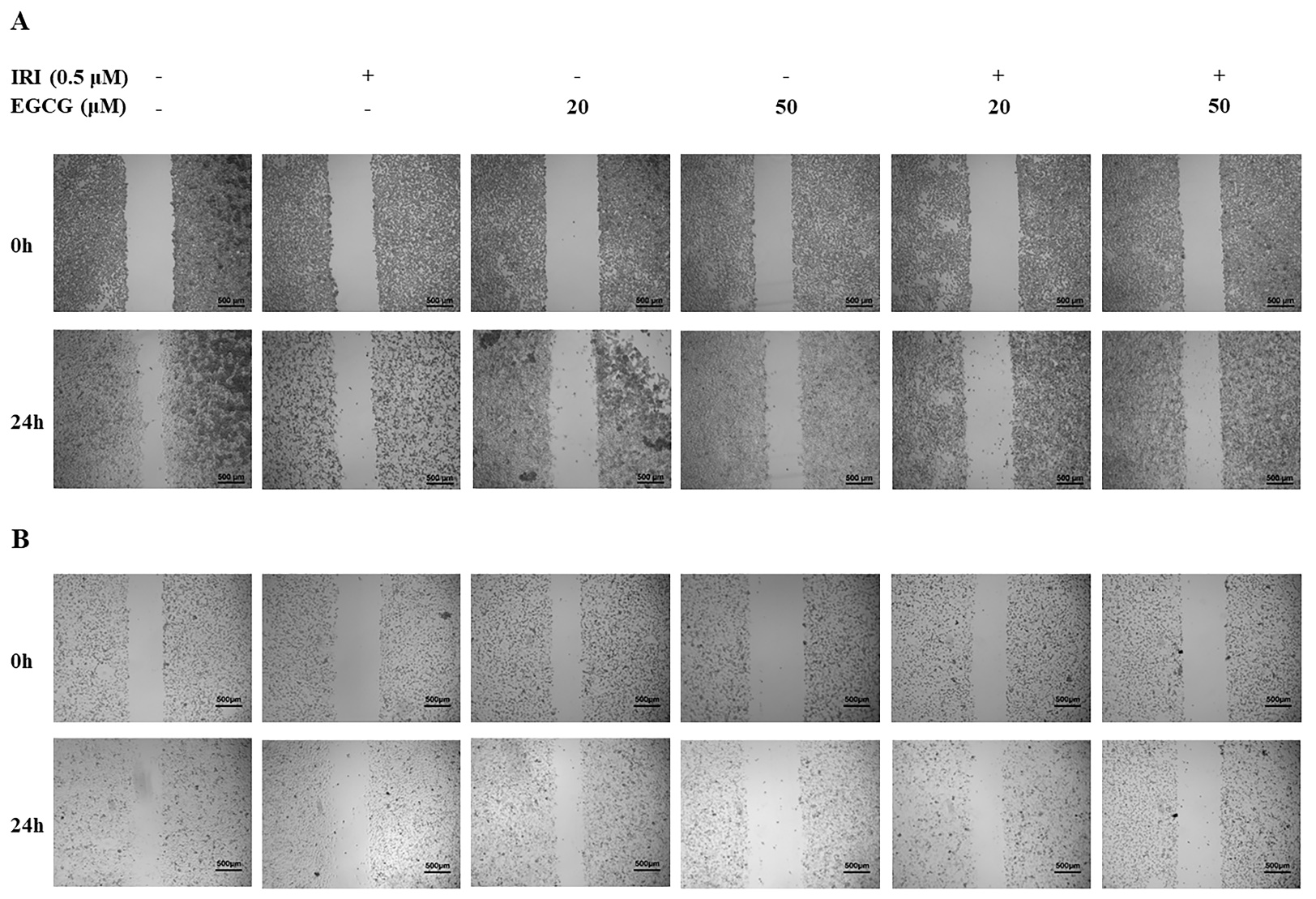
**FIGURE S1** Wound healing assay to detect the inhibitory effect of irinotecan and/or EGCG treatment on migration of RKO (A) and HCT116 (B) cells.

**
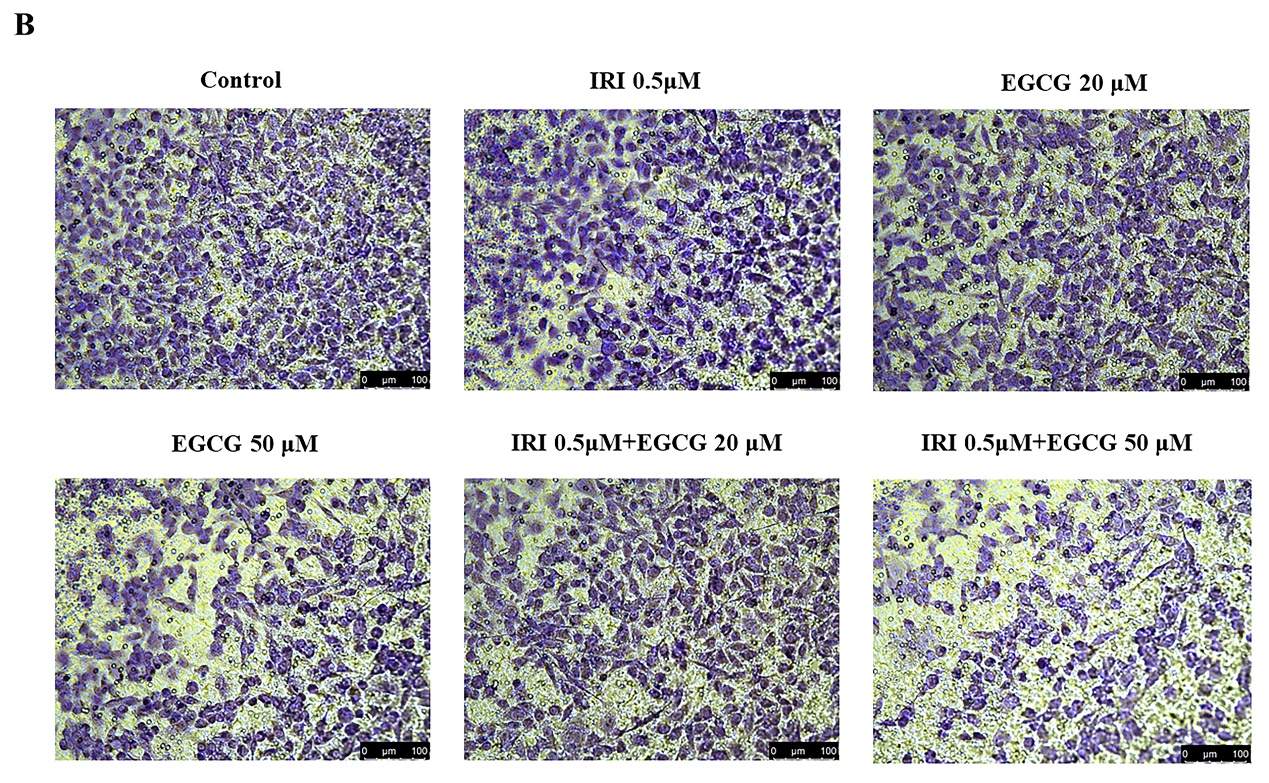
FIGURE S2** Wound healing assay to detect the inhibitory effect of irinotecan and/or EGCG treatment on migration of HCT116 cells.


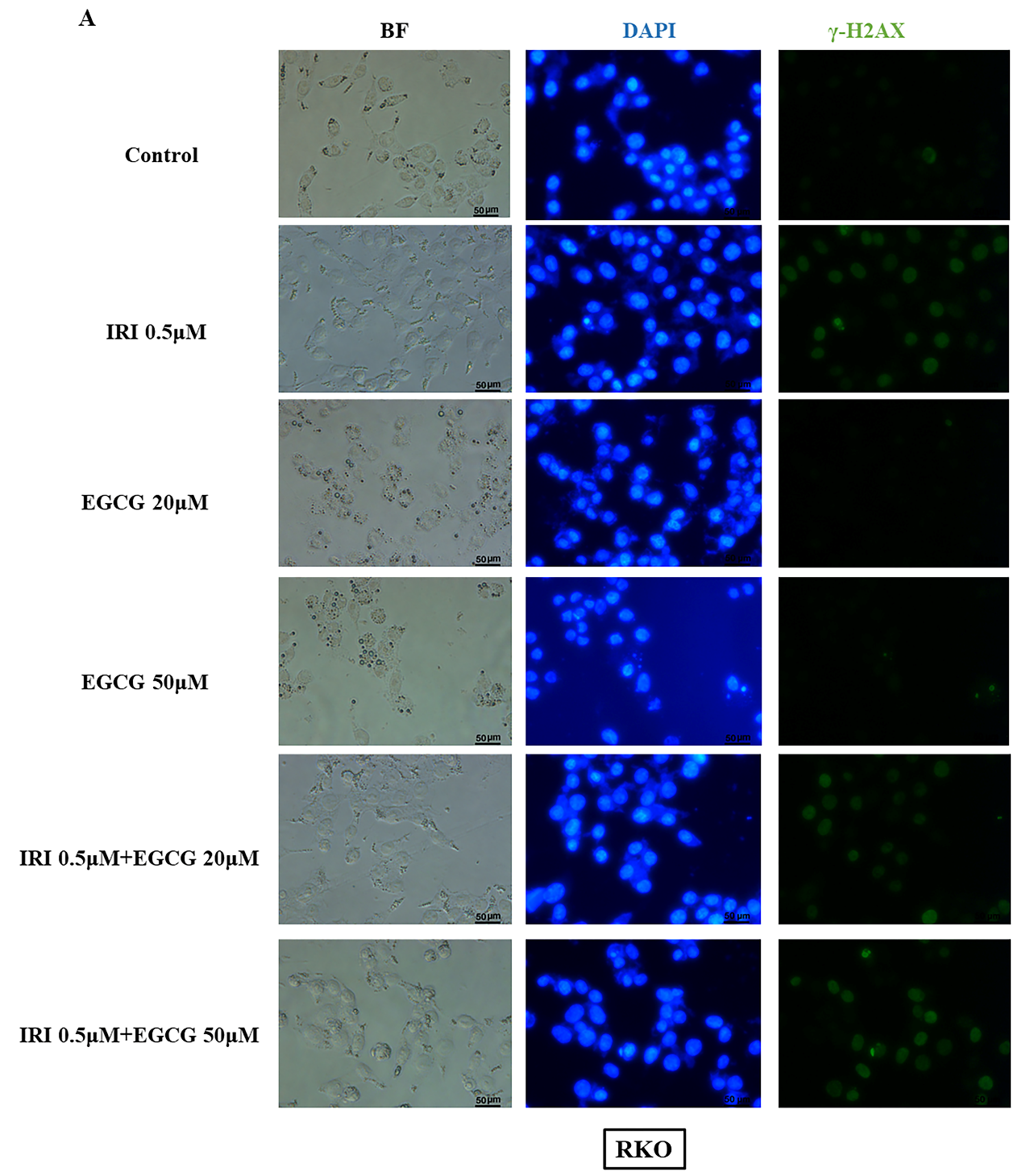


**
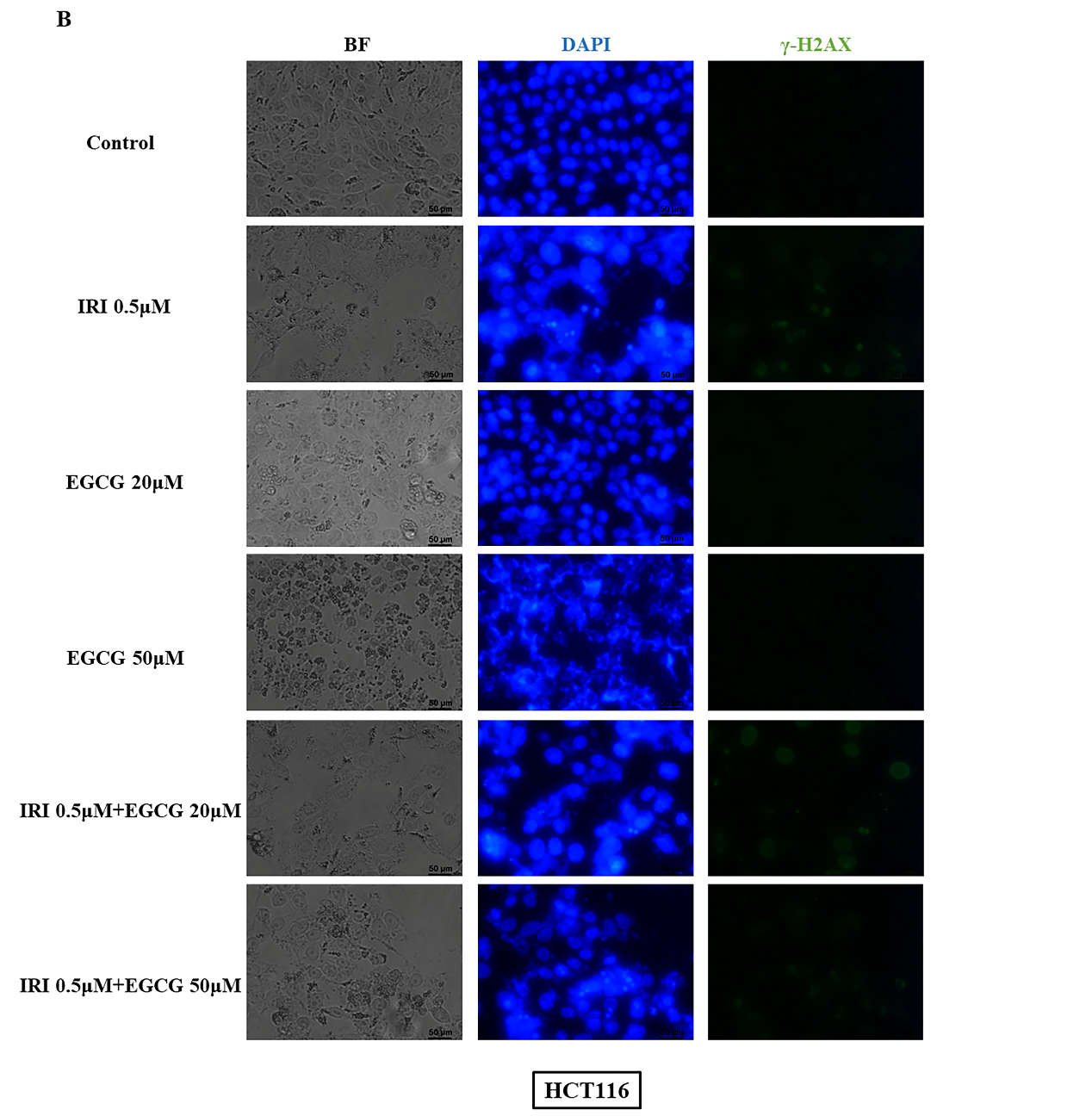
FIGURE S3** Immunofluorescence assay showed that EGCG combined with irinotecan enhanced the expression of γ-H2AX, a DNA damage marker, in RKO (A) and HCT116 (B) cells.


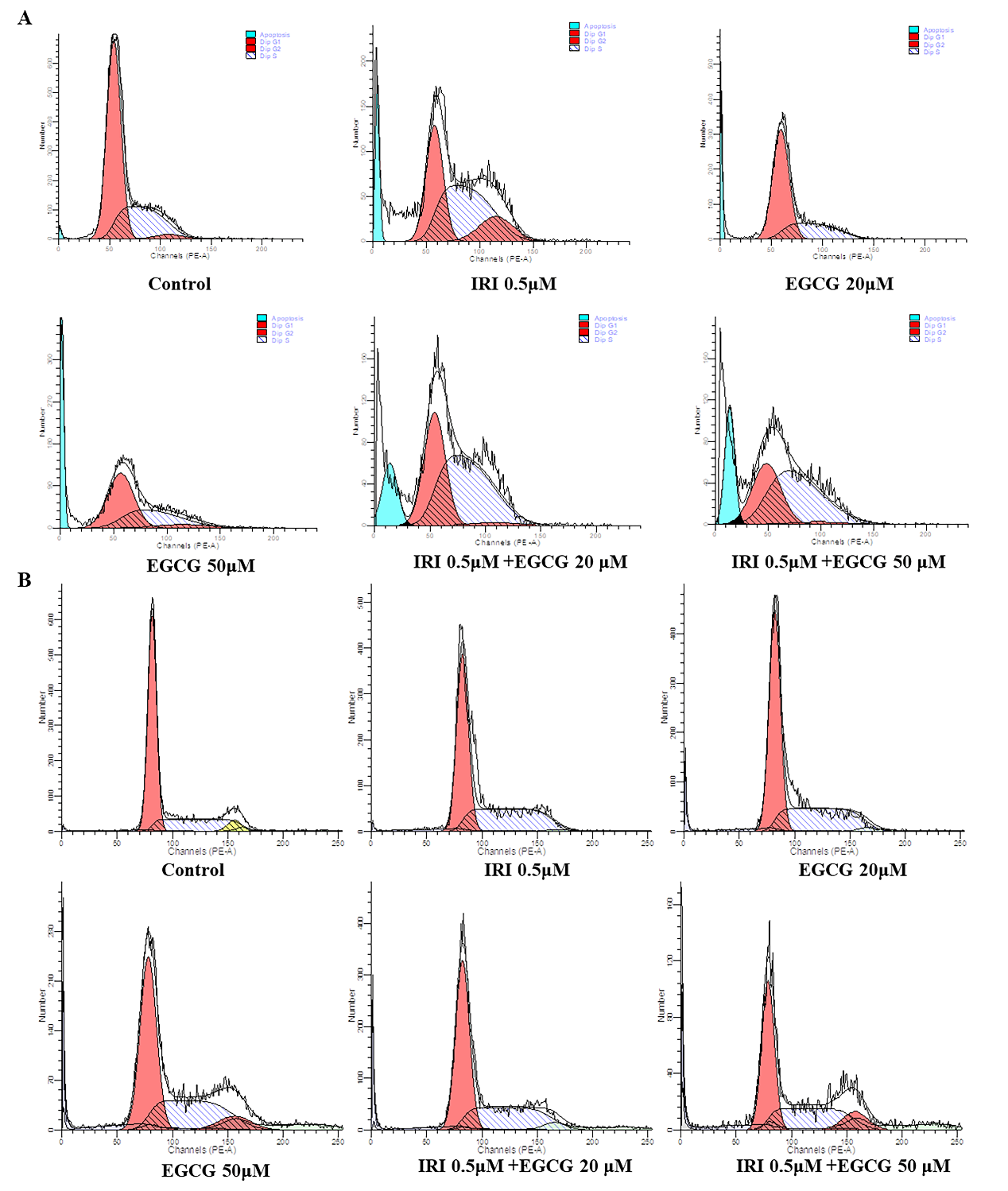


**FIGURE S4** The cell cycle distribution of RKO (A) and HCT116 (B) was tesed by propanidine staining and fitted with Modfit software.

**
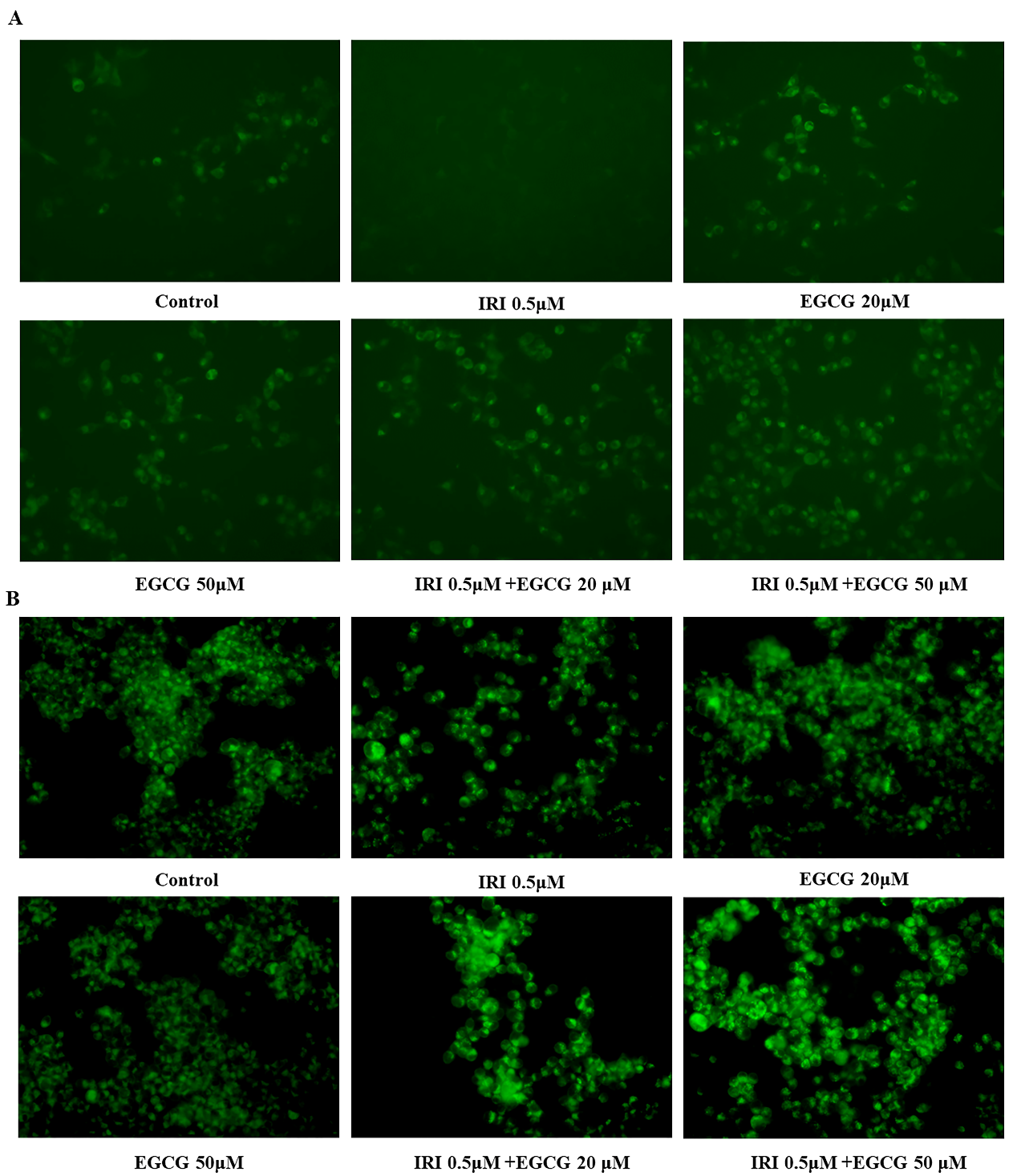
FIGURE S5** MDC staining was used to observe the autophagy vesicles of RKO (A) and HCT116 (B) cells treated by EGCG combined with irinotecan.

**FIGURE S6** After MDC staining, autophagy changes in RKO (A) and HCT116 (B) cells were detected by flow cytometry
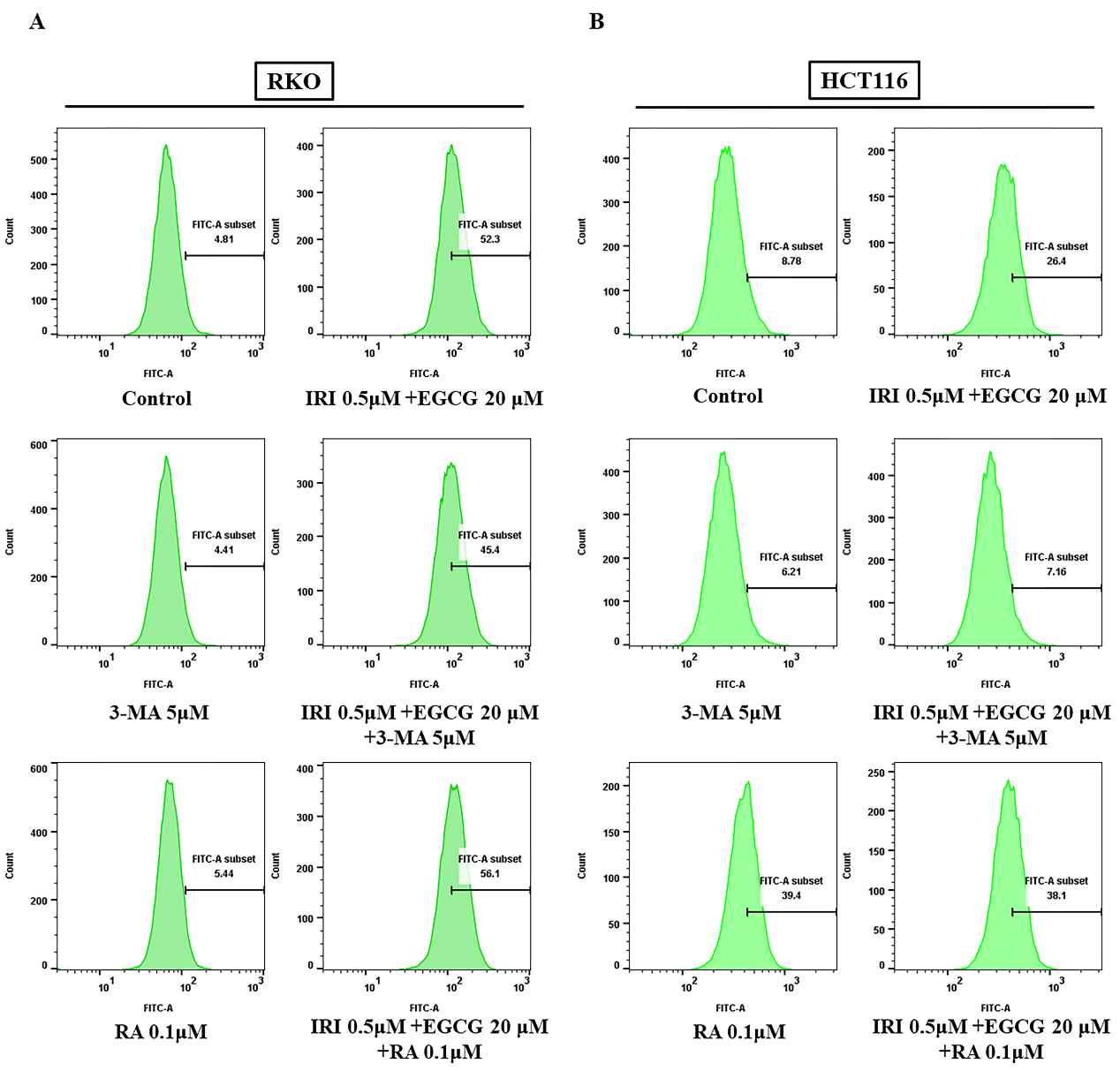
.

**
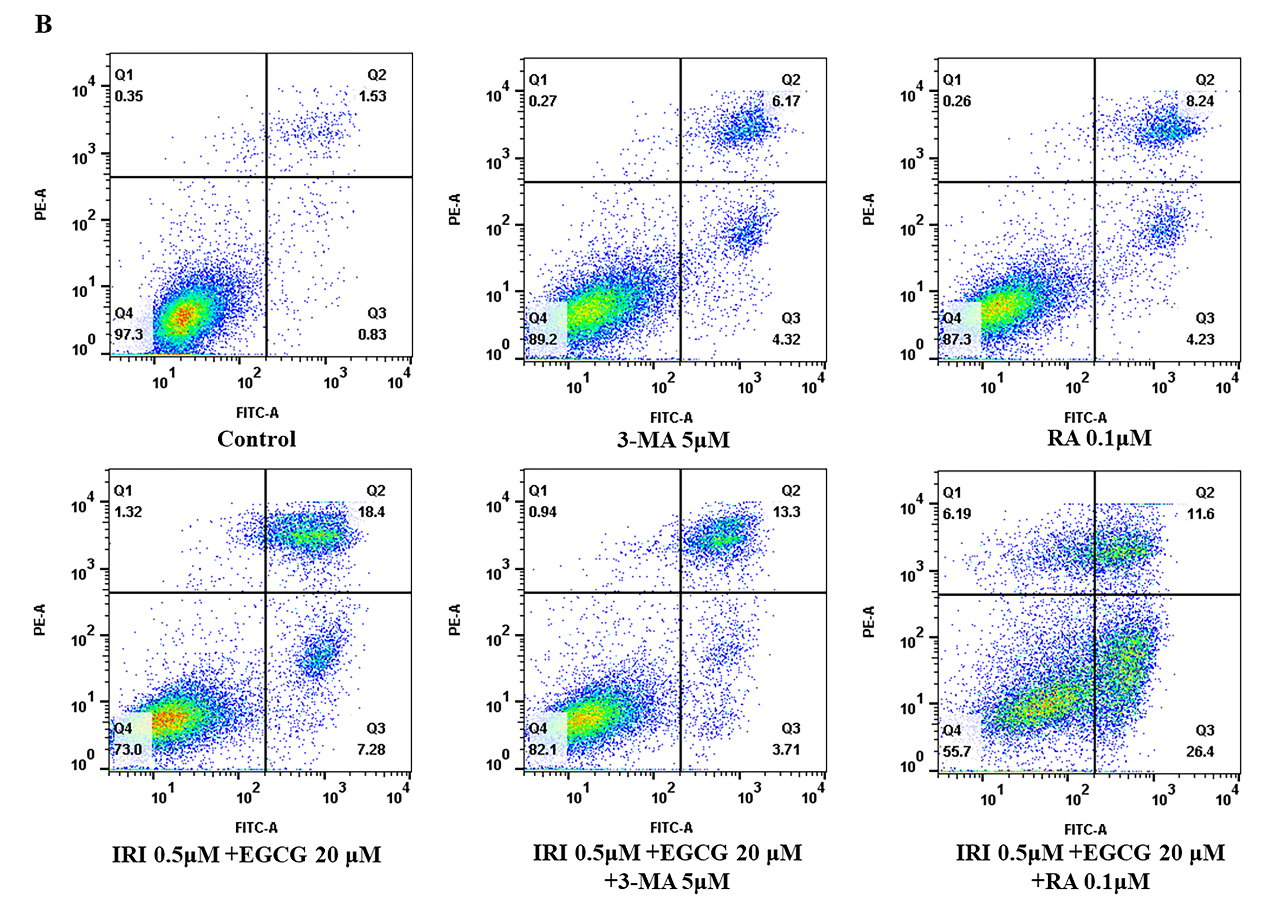

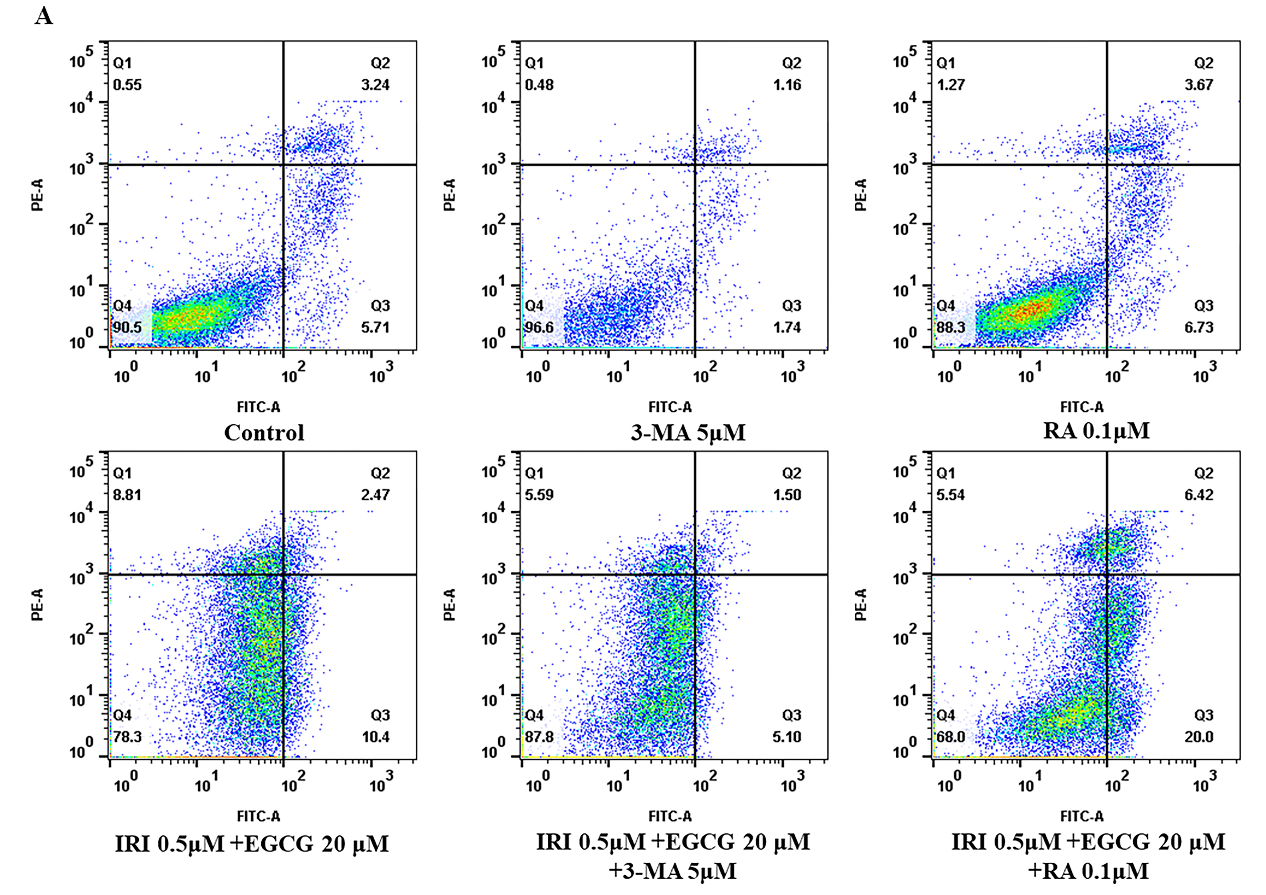
FIGURE S7** Annexin V-FITC&PI double staining was applied to test apoptosis rate of RKO (A) and HCT116 (B) cells, and the data were fitted with FlowJo software.
